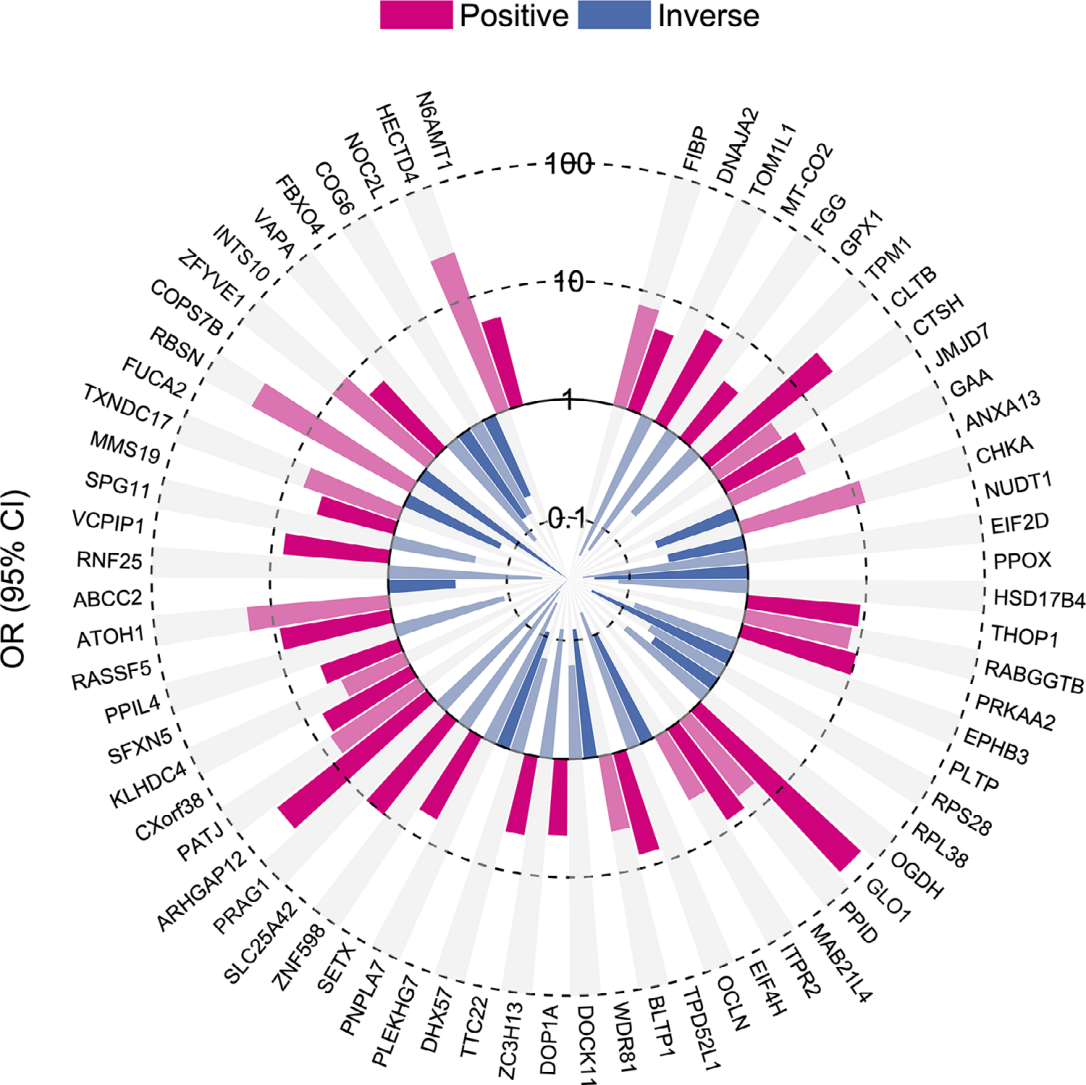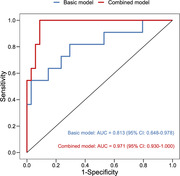# Tear proteomic signature associated with mild cognitive impairment and dementia: an exploratory analysis

**DOI:** 10.1002/alz.089992

**Published:** 2025-01-09

**Authors:** Changzheng Yuan, Jie Shen, Yuhui Huang, Minyu Wu, Yan Zheng, Tiannan Guo, Xin Xu

**Affiliations:** ^1^ School of Public Health, the Second Affiliated Hospital, Zhejiang University School of Medicine, Hangzhou, Zhejiang China; ^2^ Harvard T.H. Chan School of Public Health, Boston, MA USA; ^3^ Zhejiang University, Hangzhou, Zhejiang province China; ^4^ State Key Laboratory of Genetic Engineering, Human Phenome Institute, and School of Life Sciences, Fudan University, Shanghai China; ^5^ Westlake University, Hangzhou, Zhejiang China; ^6^ School of Public Health and the Second Affiliated Hospital of School of Medicine, Zhejiang University, Hangzhou, Zhejiang China

## Abstract

**Background:**

Tear samples were low‐invasive to access and may reflect changes in the brain. We performed an exploratory proteomic analysis using tear samples to identify proteomic signature and potential pathways that may be associated with mild cognitive impairment (MCI) and dementia.

**Method:**

We performed a matched case‐control study using tear samples collected from community‐dwelling older adults, comprising 13 dementia, as well as 34 MCI and 34 age‐, sex‐, educational‐matched normal cognition (NC) controls (age: 73.3 ± 7.1 years; female: 68.4%). The disease staging has been distinguished by clinical dementia rating (CDR). PulseDIA‐based (gas phase fractionation‐assisted data‐independent acquisition mass spectrometry) proteomics analysis was performed. Logistic regression models were used to identify differential proteins when comparing dementia versus NC and conditional logistic regression models were used when comparing MCI versus NC. Identified differential proteins were used to perform enrichment analysis. Lasso regression models were utilized to select protein biomarkers for dementia prediction.

**Result:**

A total of 70 tear proteins were found to be significantly associated with dementia and demonstrated same directions in relation to MCI. In particular, 37 proteins were positively related to dementia, with OR ranging from 3.79 (KLHDC4) to 71.91 (GLO1); 33 proteins were inversely related to dementia, with OR ranging from 0.27 (ABCC2) to 0.03 (COPS7B). The 3 strongest pathways identified by the protein‐protein interaction enrichment analysis were central nervous system neuron development, Eukaryotic Translation Termination, and amide biosynthetic process. In addition, incorporating 13 protein biomarkers to the traditional risk factors‐based model significantly improved the predictive ability of dementia (AUC from 0.813 to 0.971, P = 0.032).

**Conclusion:**

Differential tear proteins between dementia and normal cognition were identified in this exploratory analysis, which may contribute to discovering novel and low‐invasive biomarkers of dementia.